# Translation, cross-cultural adaptation, validity, and reliability of the Indonesian version of the Oxford Shoulder Score for patients with shoulder pain

**DOI:** 10.1016/j.jseint.2024.08.175

**Published:** 2024-08-24

**Authors:** Romy Deviandri, Afrianto Daud, Iman W. Aminata, Putri Octarina, Nasywa D. Mecca, Hugo C. van der Veen, Inge van den Akker-Scheek

**Affiliations:** a Department of Surgery, Faculty of Medicine, Universitas Riau, Arifin Achmad Hospital, Pekanbaru, Indonesia; b Department of Orthopedics, University of Groningen, University Medical Center Groningen, Groningen, The Netherlands; c Division of Sports Injury, Fit Centre Institute, Pekanbaru, Indonesia; d Faculty of Teachers Training and Education, Universitas Riau, Pekanbaru, Indonesia; e Department of Orthopaedic and Traumatology-Fatmawati Hospital, Jakarta, Indonesia

**Keywords:** Outcome, Psychometrics, Patient-reported, Questionnaire, Shoulder, Quality of life

## Abstract

**Background:**

No questionnaire is currently available for use in patients with shoulder pain in an Indonesian-speaking population. This study aimed to translate the Oxford Shoulder Score (OSS) into Indonesian and assess its validity and reliability for use in Indonesian-speaking patients with shoulder pain.

**Methods:**

After a forward and backward translation procedure, the validity and reliability of the questionnaire were investigated. All patients who were treated in a hospital in Indonesia for shoulder pain during the inclusion period were asked to complete 3 questionnaires: the Indonesia-OSS (I-OSS), the Medical Outcomes Study 12-Item Short-Form Health Survey, and the American Shoulder and Elbow Surgeons questionnaire. Participants were asked to complete the I-OSS a second time after a 1-week interval. Following Consensus-Based Standards for the Selection of Health Measurement Instruments guidelines, construct validity, test-retest reliability, internal consistency, floor and ceiling effects, and measurement error were determined. The Bland-Altman method was used to explore systematic bias.

**Results:**

Data of 100 patients could be used to determine validity, and data of 87 patients to determine test-retest reliability. Construct validity can be considered good, as more than 75% of the predefined hypotheses on correlations between the I-OSS and the other questionnaires could be confirmed. An intraclass correlation coefficient value of 0.99 was found, indicating good test-retest reliability. A Cronbach’s α of 0.95 implied good internal consistency, and no floor or ceiling effects were found. The standard error of measurement was 1.8, with minimal detectable change at the individual level was 5.1, and at the group level was 0.5. Bland-Altman analysis showed no systematic bias.

**Conclusion:**

The I-OSS can be considered a valid and reliable questionnaire for Indonesian-speaking patients with shoulder pain.

Shoulder pain with overhead activity and limited range of motion are common symptoms of shoulder disease reducing the quality of life of patients. The community prevalence of shoulder pain varies widely across countries, with a median of 16% (range 0.67%-55.2%). The number of persons with shoulder pain is quite vast, with the incidence of shoulder pain ranging from 7.7 to 62 per 1000 persons per year.^18^ Rotator cuff disease is one of the common causes of persistent shoulder complaints.^8^^,^^20^ It is associated with pain and decreased shoulder mobility, functional abilities, work activities, and quality of life.^2^ Measurement tools are essential and critical in patient management to appraise subjective health and evaluate and improve therapeutic measures.^15^^,^^33^

Many measurement methods have been developed over the years to assess the outcome of treatment of shoulder diseases; among these are patient-reported outcome measures (PROMs).^6^^,^^21^ PROMs can be general or investigating a specific disease, whereby the patient’s perspective of their pain, function, daily life problems, and quality of life, for example, is assessed.^15^^,^^31^ As most questionnaires reflect characteristics of the population in which they were established, such as language and social culture, translation and cultural adaptation of PROMs are vital for use in other communities.^7^^,^^33^

Published in 1996 and modified in 2009 by Dawson et al, the Oxford Shoulder Score (OSS) is a widely used shoulder-specific PROM for assessing patients with shoulder pain.^9^^,^^10^ OSS is a self-assessment of pain and function of the shoulder, which is considered quick, simple, and reliable.^29^ Because the OSS was originally developed in an English-speaking country, it needs a translation and cross-cultural adaptation process before being used in populations with other languages and cultures. Moreover, the clinimetric properties of these versions should again be assessed. The OSS questionnaire has already been translated and adapted into different languages but not into Indonesian,^3^^,^^4^^,^^8^^,^^11^^,^^15^^,^^22^^,^^23^^,^^27^, ^28^, ^29^^,^^33^ whereas the Oxford hip and knee scores have already been validated in Indonesian.^24^

This study aims to translate and cross-culturally adapt the original OSS into an Indonesian-language version following internationally accepted guidelines^19^ and assess the validity and reliability of this Indonesia-OSS (I-OSS).

## Methods

An institutional review board approved the study protocol, and patients provided informed consent before participating in this study. The developers of the OSS approved the translation procedure.

### Translation procedure

**Figure 1:**
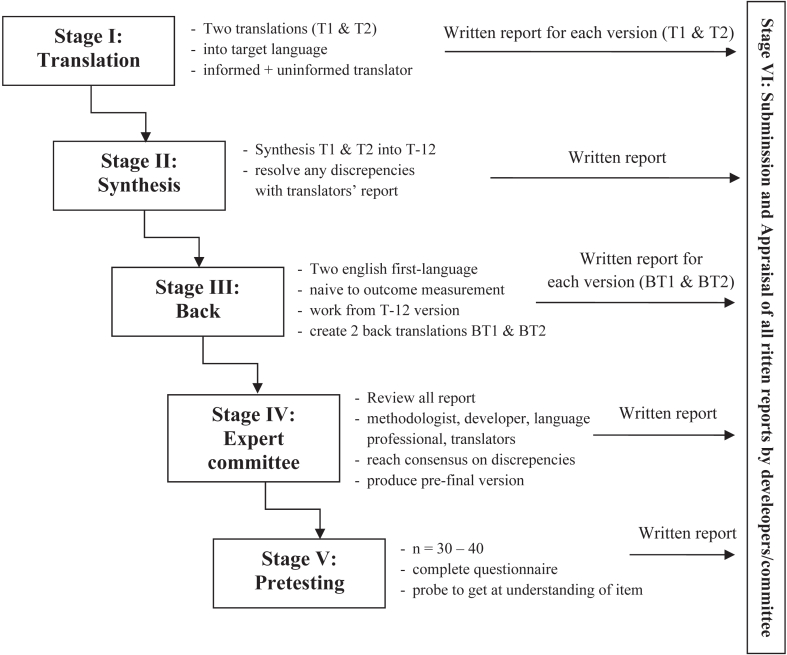
Flowchart of the translation procedure.^2^^,^^12^

The I-OSS translation follows the guidelines for both forward and backward translation, which were first introduced by Guillemin et al and modified by Beaton et al.^2^^,^^12^ The primary process comprises 5 stages: initial translation, synthesis, back-translation, expert committee review, and pretesting. The initial translation stage was started by 2 independent Indonesian-English licensed translators who completed the conceptual and literal translation of OSS into Indonesian (T1 and T2). Next, the synthesized version (T12) was created using feedback from the 2 initial translations. T12 was then independently translated back into English by 2 licensed Indonesian-English translators (BT1 and BT2) to identify any inconsistencies with the original English version. Subsequently, a committee of experts, comprising 3 orthopedic surgeons, 1 methodologist, and 1 translator, reviewed the Indonesian translation. Following the committee’s review, one of the authors (R. D.) edited the questionnaire to create a prefinal translated version. Then, the pretesting stage was conducted by distributing the prefinal translated version to 10 patients with shoulder problems. One researcher (R. D.) documented difficulties experienced by the patients when completing the questionnaire. Documentation was reviewed to modify the questionnaire into the final version of the I-OSS (Fig. 1).

### Patients and procedure

We included all Indonesian-speaking patients treated for shoulder pain—diagnosis validated by an orthopedic surgeon who has experienced more than 5 years in this field—between July 2022 and June 2023 at a hospital in Indonesia. Subjects were selected after obtaining formal informed consent and checking the inclusion and exclusion criteria. To be included in this validation study, subjects had to speak Indonesian as a primary language, complain of shoulder pain, and were treated for shoulder pain in the hospital. The patients with neuropathic pain, neoplasms, systemic inflammatory diseases, and shoulder instability were excluded. After informed consent, patients were given a first set of questionnaires (part A) to be filled out at the outpatient clinic. Then, patients were asked to come again after 1 week and fill out a second set of questionnaires (part B).

Part A consisted of the I-OSS and 2 complementary questionnaires, the Medical Outcomes Study 12-Item Short-Form Health Survey (SF-12) and the American Shoulder and Elbow Surgeons (ASES) questionnaire, to be used to assess validity. Part B consisted of only a single questionnaire, the I-OSS, to assess test-retest reliability. Additionally, to determine whether the patient’s health status and shoulder function remained stable between the completion of parts A and B, patients were provided at the start of the part B questionnaire with a Global Rating of Change (GRC) question: “Has your status changed since filling out the initial questionnaire?” There were 3 potential responses: (1) no change, (2) improvement in the problem, and (3) worsening of the problem. Only patients who reported no change in their shoulder symptoms were included in the test-retest analysis. Moreover, those patients who completed the second set of questionnaires more than 1 month later were excluded.

### Patient-reported outcome measures

The OSS is a shoulder-specific questionnaire for assessing patients with shoulder pain. It has 12 items, consisting of 4 items about pain and 8 questions about daily shoulder function. Each item is assessed based on 5 categories of answers, which are 0 (worst) to 4 (best condition), and a total score ranging from 0 to 48.^9^^,^^10^

The ASES score is a shoulder-specific questionnaire. This questionnaire comprised of 11 questions, which are separated into 2 sections. The first section, focusing on pain, assesses the patient’s level of pain using a 10-cm visual analog scale. This scale ranges from 0, indicating no pain, to 10, representing the most severe pain. The second section, function, comprises 10 questions that assess the capacity to carry out everyday tasks, from simple, such as putting a coat, to more demanding, such as tossing a ball above one’s head or raising a 10-pound item. Answers are on a 4-point Likert scale from 0, unable to do, to 3, not difficult. The scores derived from the pain and function subsections are converted into percentages, with each accounting for 50% of the total score. Scores on the ASES range from a minimum of 0 points to a maximum of 100 points. We translated the ASES into Indonesian following the international guidelines.^32^

The SF-12 is a generic PROM assessing health-related quality of life. It consists of 8 subscores: physical functioning, physical role functioning, bodily pain, general health perceptions, vitality, social function, emotional role functioning, and mental health. In addition, the sum of the physical functioning, physical role functioning, bodily pain, and general health perceptions subscales generates a physical component summary score (PCS), and the sum of the vitality, social function, emotional role functioning, and mental health subscales generates a mental component summary score (MCS). Standardized scores range from 0 to 100, with higher scores indicating better health status.^30^ SF-12 was translated to Indonesian version and shown a reliable and valid measure of health-related quality of life in Indonesian middle-aged and older adults.^1^

### Validity

Validity refers to the degree to which a measurement instrument accurately measures what it intends to measure, ensuring it aligns with the proposed interpretation. The construct validity of the I-OSS was assessed by determining the correlation of the I-OSS total score with the total score on the ASES, the PCS, and the MCS. In line with the Consensus-Based Standards for the Selection of Health Measurement Instruments (COSMIN) guidelines, predetermined hypotheses were defined regarding the strength of the correlation between the I-OSS and the ASES, PCS, and MCS.^19^ Since both I-OSS and ASES questionnaires are shoulder-specific PROMs, we hypothesized that the I-OSS would correlate better with the ASES than with the PCS and MCS. The correlation between the original OSS and the ASES was 0.91 in a previous study.^13^ Based on the correlations found in that study, correlations of 0.6 or more between the I-OSS and the ASES were hypothesized. The Turkish version of OSS showed a correlation of 0.63 with the PCS and 0.38 with the MCS. Based on this, it was hypothesized to find correlations of 0.5 or more between the I-OSS and the PCS and the correlation less than 0.5 between the I-OSS and the MCS. The I-OSS was designed primarily to assess physical functioning, as opposed to social or emotional aspects. Consequently, stronger correlations were anticipated between the I-OSS and the PCS compared to those between the I-OSS and the MCS. Good construct validity is indicated when a minimum of 75% of these hypotheses are verified.^26^

### Floor and ceiling effects

The presence of floor and ceiling effects was evaluated. These effects are deemed to exist if more than 15% of the participants attain either the lowest or highest possible score on the I-OSS.^26^

### Reliability

Reliability reflects the degree to which individuals can be differentiated from one another in spite of errors in measurement. In accordance with the COSMIN guidelines, reliability was evaluated through 3 aspects: internal consistency, test-retest reliability, and measurement error.^19^^,^^26^ Internal consistency is about how closely related the items of a questionnaire are to each other. Test-retest reliability focuses on how consistent patients’ scores remain across repeated measurements in a period where no actual change in the construct that is being measured is expected. Measurement error, on the other hand, quantifies the systematic inaccuracies in a patient’s score that are not due to actual changes in the construct being measured. To assess systematic bias, the Bland-Altman method was employed.^5^

### Statistical analysis

The characteristics of the study population and scores on the questionnaires are described using means and standard deviations, or frequencies and percentages. To determine construct validity, Spearman rho correlation coefficients were calculated between the scores on the I-OSS and the other questionnaires. The Spearman rho values were interpreted as high (r > 0.6), moderate (0.6 < r < 0.3), or low (r < 0.3).^13^ To determine internal consistency, Cronbach’s α was calculated. Values between 0.70 and 0.95 indicate good internal consistency.^26^

To evaluate the test-retest reliability, the intraclass correlation coefficient (ICC) between the initial and subsequent I-OSS scores was calculated. Values < 0.5, 0.5-0.75, 0.75-0.9, and > 0.90 indicate poor, moderate, good, and excellent reliability, respectively.^16^^,^^17^ To assess measurement error, the standard error of measurement (SEM) and the minimal detectable change (MDC) were calculated. SEM was determined by multiplying the pooled standard deviation by √(1-*r*), where *r* is the ICC. The MDC at the individual level (MDC_ind_) was determined using the formula 1.96 × SEM × √2 and MDC at the group level (MDC_grp_) by dividing MDC_ind_ by √n.^14^^,^^26^

Absolute reliability was evaluated using Bland-Altman plots. The absence of systematic bias was indicated when 0 fell within the 95% confidence interval (CI) of the mean difference between the first and second administration of the I-OSS. The 95% limits of agreement were measured with the formula mean difference ± 1.96 × SD_diff_, where SD_diff_ is the standard deviation of the mean difference between the first and second administration of the I-OSS.^4^ Statistical analyses were conducted using SPSS Statistics version 26.0 (IBM Corp., Armonk, NY, USA), with a level of significance set at 5%.

## Results

### Patient characteristics

In total, 100 patients completed part A, and their responses were used to study validity and internal consistency. Ninety-two patients (92%) additionally also filled in part B. Five patients were excluded as they reported better shoulder function when they filled out part B compared to when they completed part A. No patients were excluded due to missing data. Thus, to assess test-retest reliability, data from 87 patients were used. Most of the patients graduated from high school, and the mean age was 50.3 ± 12.9 years. The details of the demographic characteristics of the patients are provided in Table I.

**Table I:** Demographic characteristics of the participants (N = 100).

Characteristic	Mean ± SD or n (%)
Age (y)	50.3 ± 12.9
Sex	
Male	28 (28)
Female	72 (72)
Diagnosis	
Adhesive capsulitis	23 (23)
Rotator cuff tear	68 (68)
Shoulder impingement	9 (9)
Affected site	
Right	59 (59)
Left	41 (41)
Education	
Primary school	1 (1)
Middle school	5 (5)
High school	63 (63)
University	31 (31)

*y*, year; *SD*, standard deviation.

Mean scores of the first and second assessments of the I-OSS were 33.9 ± 11.0 and 33.8 ± 11.4, respectively. All scores on the PROMs can be found in Table II.

**Table II:** Scores on patient-reported outcome measures.

Measure	Mean ± SD
I-OSS	
Part A	33.9 ± 11.0
Part B	33.8 ± 11.4
ASES	49.4 ± 16.3
SF-12	
PCS	32.7 ± 3.5
MCS	33.8 ± 4.5

*SD*, standard deviation; *I-OSS*, Indonesia-Oxford Shoulder Score; *ASES*, American Shoulder and Elbow Surgeons score; *SF-12*, Medical Outcomes Study 12-Item Short-Form Health Survey; *PCS*, physical component summary score; *MCS*, mental component summary score.

### Validity

Of the 6 predefined hypotheses on the magnitude of associations between the I-OSS and the ASES, PCS, or MCS, 5 hypotheses were confirmed (a total of 83%). The I-OSS showed strong correlations with the ASES (*r* = 0.7), low correlation with the PCS (*r* = 0.3), and low correlation with MCS (*r* = 0.1). As hypothesized, the I-OSS was more strongly related to the ASES than to the PCS and MCS and more strongly related to PCS than to MCS (Table III). There were no floor or ceiling effects.

**Table III:** Spearman rho correlation coefficients among I-OSS, ASES, PCS, and MCS.

	Correlation	Hypothesized	Found	Confirmed
1	I-OSS and ASES	> 0.6	0.7	Yes
2	I-OSS and PCS	≥ 0.5	0.3	No
3	I-OSS and MCS	< 0.5	0.1	Yes
4	I-OSS and ASES correlation stronger than that between I-OSS and PCS		0.7:0.3	Yes
5	I-OSS and ASES correlation stronger than that between I-OSS and MCS		0.7:0.1	Yes
6	I-OSS and PCS correlation stronger than that between I-OSS and MCS		0.3:0.1	Yes

*I-OSS*, Indonesia-Oxford Shoulder Score; *ASES*, American Shoulder and Elbow Surgeons score; *MCS*, mental component summary score; *PCS*, physical component summary score.

### Internal consistency, test-retest reliability, and measurement error

**Figure 2:**
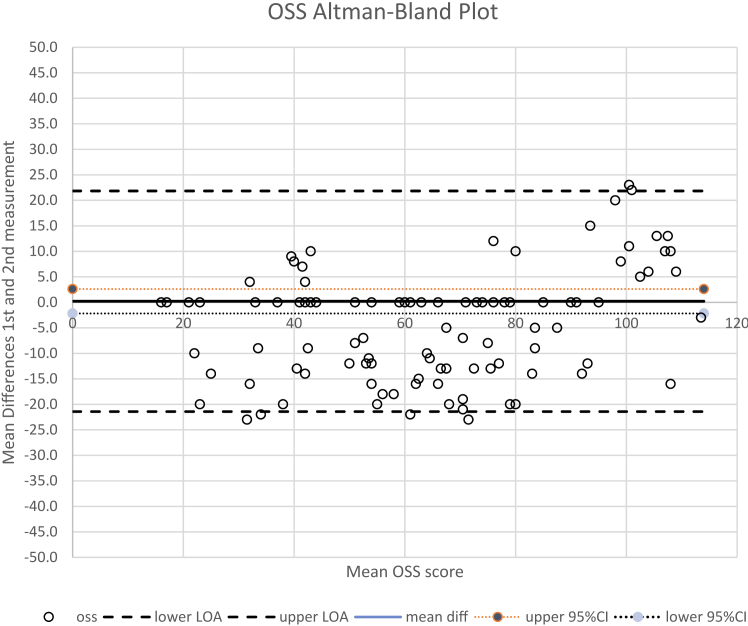
The Bland-Altman plot illustrates the mean difference between the first and second measurements of the Indonesian version of the OSS. In this plot, *open circles* depict individual data points. The mean difference is shown by a *solid black line*. The *dotted lines* indicate the 95% confidence intervals (CIs), and the *dashed lines* represent the limits of agreement. *OSS*, Oxford Shoulder Score.

The Cronbach’s α was 0.95, indicating good internal consistency. The ICC had a value of 0.99 (*P* < .001), and the 95% CI ranged from 0.997 to 0.999. The SEM, MDC_ind_, and MDC_grp_ were determined to be 1.8, 5.1, and 0.5, respectively. The Bland-Altman analysis showed that the average difference of the 2 I-OSS assessments was 0.2 (95% CI, −2.2 to 2.6, 95% limits of agreement, −21.4 to 21.8) (Fig. 2). No systematic bias was present because the value of 0 was in the 95% CI of the mean difference between the test and retest scores.

## Discussion

This study aimed to translate the OSS into Indonesian and to gain insight into the validity and reliability of this questionnaire in an Indonesian-speaking population with shoulder pain. Based on the results, OSS can be considered valid and reliable for use in Indonesian-speaking patients.

The construct validity of I-OSS can be considered good, with more than 75% of predefined hypotheses confirmed. As hypothesized, I-OSS showed a strong correlation with ASES (r = 0.7). This is comparable with a previous study that showed a good correlation between the 2 (r = 0.91).^13^ As hypothesized, I-OSS showed a low correlation with the MCS (r = 0.1). This result is in line with the study on the Turkish version of the OSS (r = 0.38).^28^ Moreover, the I-OSS was more strongly correlated to the PCS domains than to the MCS domains, again confirming our hypothesis. However, contrary to our hypothesis, the correlation of the I-OSS with PCS was less than 0.5. This was probably caused by the difference in characteristics of the participants and also the variation in diseases of the subjects among those studies. A cultural difference also might be correlated with the relatively poor validity noted between the PCS and I-OSS. According to prior study, there is a cultural difference between European population and Indonesian population regarding the PCS component of SF-12 where PCS is negatively affected more than MCS as we age in Indonesian population than in European population.^1^ However, I-OSS was more strongly correlated to the PCS domains than to the MCS domains, confirming our hypothesis.

No floor or ceiling effects were observed. Theoretically, a ceiling effect could have occurred, especially in patients whose adhesive capsulitis was healing and had taken physiotherapy adequately, but this was not the case. No floor or ceiling effects were found in other-language versions, like Persian and Turkish.^22^^,^^28^ This result indicates a good content validity of I-OSS. Also, the internal consistency of I-OSS can be considered excellent, with a Cronbach’s α of 0.95. This is comparable with the Chinese (0.92), Dutch (0.92), German (0.94), Turkish (0.92), and Persian (0.91) versions.^4^^,^^15^^,^^22^^,^^28^^,^^33^

The I-OSS showed excellent test-retest reliability (ICC, 0.99). This is comparable with the Polish (0.99), Chinese (0.97), Dutch (0.98), Turkish (0.99), and Persian (0.90) versions [3,5,6,16,17].^4^^,^^8^^,^^15^^,^^28^^,^^33^ All showed excellent test-retest reliability with the test-retest time interval of 1 to 2 weeks. In accordance with COSMIN guidelines, the interval between the test and retest should be sufficiently long to avoid recall bias, yet short enough to ensure that the patients’ characteristics relevant to the construct being measured remain unchanged.^18^^,^^25^ In this study, a week of time interval was used. Also, this study used the GRC question as an exclusion for patients that actually changed, which may have resulted in a high ICC value compared to some other studies. The application of GRC in the reliability study is a good practice and results in better insight into reliability.

SEM of I-OSS was 1.8, MDC_grp_ was 0.5, and MDC_ind_ was 5.1. These values are comparable to those presented in the Polish study of the OSS (SEM 1.14, MDC_ind_ 3.15).^8^ The results indicated sufficient capability of the I-OSS for comparisons at group level, as only small values are needed to detect change. As only values greater than the SEM can be reliably differentiated from the measurement error, a difference should exceed 1.8 to detect a statistically significant change in scores on I-OSS at group level. The difference between the 2 measurements should be greater than the MDC_ind_ value in individual patients and SEM, so it can be distinguished from the measurement error and to confirm a real change occurred. Given that the MDC_ind_ is quite low (5.1), this indicates sufficient capability of I-OSS as an appropriate tool for monitoring individual patients over time.

### Limitations

There are some limitations to this study. There were some patients who did not come in the second time, yet the total number of participants is deemed adequate: following COSMIN guidelines, at least 100 patients are required to assess validity and 50 patients to examine test-retest reliability.^25^ Furthermore, this population is only comprised of patients with rotator cuff disease, adhesive capsulitis, and shoulder impingement. Other possible populations, such as glenohumeral arthritis diagnoses, were not included in the present study. This may make the findings in this study less generalizable to other populations.

Another potential limitation is that the comparison of the I-OSS was made with only one other disease-specific questionnaire, the ASES, and a general health-related quality of life questionnaire (SF-12). However, no other Indonesian versions of a PROM are available to assess shoulder function or patients’ quality of life until now. Consequently, the number of hypotheses was relatively less.

Next, future research should explore the responsiveness of I-OSS as well as minimal clinically important difference (MCID). MCID should be determined to examine further whether a measured difference is also clinically important as perceived by the patient. For the Danish OSS version, the reported MCID was 6.0 with an effect size of 0.96.^16^^,^^25^ These findings are not transferable to the Indonesian version though, so the responsiveness and MCID of I-OSS should be assessed in future research. The information on responsiveness and capability of I-OSS to detect change over time is required for the interpretation of these scores when used in longitudinal research and in clinical practice to monitor Indonesian-speaking patients over time.

## Conclusion

I-OSS can be considered a valid and reliable questionnaire for use in Indonesian patients with shoulder disease. This questionnaire enables us to measure patient-perceived symptoms, function, and outcome of treatment in the Indonesian population with shoulder disease. More research is needed into MCID and responsiveness of the I-OSS.

## Acknowledgments

The authors thank all Indonesian Orthopedic Society for Sports Medicine and Arthroscopy (IOSSMA) members who participated in the study.

## Disclaimers:

Funding: No funding was disclosed by the authors.

Conflicts of interest: The authors report there are no competing interests to declare from all author, their immediate family, and any research foundation with which they are affiliated did not receive any financial payments or other benefits from any commercial entity related to the subject of this article.
